# Multiple blood pressure parameters and risk of incident diabetes in adults with elevated blood pressure: a cohort study with non-linear analyses

**DOI:** 10.3389/fendo.2026.1887291

**Published:** 2026-08-27

**Authors:** Qiaofeng Wu, Yuting Gao, Anwen Yu, Zhenhua Huang, Xin Li

**Affiliations:** 1 Department of Emergency Medicine, Guangdong Provincial People’s Hospital, Guangdong Academy of Medical Sciences, Southern Medical University, Guangzhou, China; 2 Department of Endocrinology and Metabolism, Peking University Third Hospital, Beijing, China; 3 Department of Emergency Medicine, The First Affiliated Hospital of Shenzhen University, Shenzhen Second People’s Hospital, Shenzhen, China

**Keywords:** blood pressure, Chinese population, diabetes, hypertension, non-linear association

## Abstract

**Aims:**

This study aimed to compare the strengths of association and predictive performance of systolic BP (SBP), diastolic BP (DBP), mean arterial pressure (MAP), pulse pressure (PP), pulse pressure index (PPI), and the SBP/DBP ratio for incident diabetes among Chinese adults with elevated BP.

**Methods:**

This retrospective cohort study included 29,377 adults with elevated BP from a large Chinese health examination database. Cox proportional hazards models were used to estimate hazard ratios (HRs), and restricted cubic spline analyses were applied to evaluate non-linear associations.

**Results:**

During a median follow-up of 2.98 years, 1,550 participants (5.28%) developed diabetes. After multivariable adjustment, SBP (HR: 1.09, 95% CI 1.05–1.13), MAP (HR 1.07, 95% CI 1.02–1.13), PP (HR 1.06, 95% CI 1.03–1.10), PPI (HR 2.47, 95% CI 1.36–4.46), and the SBP/DBP ratio (HR 1.38, 95% CI 1.13–1.70) were significantly associated with incident diabetes. Restricted cubic spline analyses revealed significant non-linear relationships for SBP, DBP, MAP, and the SBP/DBP ratio. Among all BP parameters, SBP demonstrated the highest predictive performance.

**Conclusions:**

Among Chinese adults with elevated BP, several BP parameters were associated with incident diabetes, and significant non-linear dose–response relationships were observed for SBP, DBP, MAP, and the SBP/DBP ratio. SBP showed the highest predictive performance, suggesting that BP parameters may provide simple supplementary information for diabetes risk stratification in this high-risk population.

## 1 Introduction

Diabetes has become a major global public health challenge. According to the International Diabetes Federation, an estimated 537 million adults were living with diabetes worldwide in 2021, and this number is projected to rise to 783 million by 2045 (1). Type 2 diabetes mellitus (T2DM) accounts for more than 90% of cases and is associated with substantial morbidity, mortality, and healthcare burden (2). In China, rapid socioeconomic transitions have led to a marked increase in diabetes prevalence, now affecting approximately 12.8% of adults, highlighting the urgent need for early identification of high-risk individuals (3).

Hypertension and diabetes frequently coexist and share common pathophysiological mechanisms, including insulin resistance, obesity, and chronic low-grade inflammation (4). Epidemiological studies have consistently demonstrated that elevated blood pressure (BP) is an independent risk factor for incident diabetes (5, 6).

However, BP is a multidimensional hemodynamic construct, and traditional measures such as systolic BP (SBP) and diastolic BP (DBP) may not fully capture its complexity. Derived parameters, including mean arterial pressure (MAP), pulse pressure (PP), pulse pressure index (PPI), and the SBP/DBP ratio—reflect distinct aspects of vascular function and arterial properties. MAP represents overall tissue perfusion pressure, while PP is widely regarded as a surrogate marker of arterial stiffness (7, 8). Increasing evidence suggests that arterial stiffness and impaired vascular function are closely linked to insulin resistance and glucose metabolism abnormalities (9). In addition, composite indices such as PPI and the SBP/DBP ratio may capture the balance between pulsatile and steady components of blood pressure, potentially providing additional information on cardiometabolic risk beyond traditional BP measures (10, 11).

Previous studies have generally shown positive associations between both systolic and diastolic blood pressure and diabetes risk, the association for SBP appears to be more consistent and stronger across studies (12, 13). Emerging evidence also indicates that PP may have independent predictive value (14). However, most existing studies have examined hypertension as a binary risk factor in general populations, and evidence directly comparing different BP parameters within individuals with elevated BP remains limited.

Although the association between hypertension and incident diabetes is well established, several important knowledge gaps remain. First, previous studies have often treated hypertension as a binary exposure or focused only on SBP and DBP (5, 15); the comparative associations and predictive performance of derived BP parameters have not been systematically evaluated. Second, emerging evidence suggests that BP may have non-linear associations with metabolic outcomes, whereas most previous analyses have assumed linearity and may therefore have missed important threshold patterns (16). Third, most studies have focused on general populations, and direct comparisons of multiple BP parameters among individuals with elevated BP, a group already at high cardiometabolic risk, remain scarce (17, 18). Addressing these gaps is clinically relevant because BP is routinely measured at little additional cost and may provide pragmatic information for risk stratification.

Accordingly, in this large cohort of Chinese adults with elevated BP, we aimed to (1) systematically evaluate and compare the associations of SBP, DBP, MAP, PP, PPI, and the SBP/DBP ratio with incident diabetes (2); use restricted cubic splines to examine potential non-linear dose–response relationships; and (3) identify the BP parameter with the highest predictive value for diabetes in this high-risk population.

## 2 Methods

### 2.1 Data sources and study population

This retrospective cohort study was based on data from the Rich Healthcare Group database, a large-scale computerized health examination platform that includes longitudinal data from multiple centers across China between 2010 and 2016 (19). The database contains comprehensive information on demographic characteristics, anthropometric measurements, laboratory tests, and medical history.

A total of 685,277 adults aged ≥20 years who underwent at least two health examinations were initially screened. Participants were excluded if they had missing data on body weight or height (n = 103,946), missing sex information (n = 1), extreme body mass index (BMI <15 or >55 kg/m², n = 152), missing fasting plasma glucose (FPG) at baseline (n = 31,370), or follow-up duration <2 years (n = 324,233). We further excluded individuals with diabetes at baseline, defined as self-reported diabetes, use of glucose-lowering medications, or FPG ≥7.0 mmol/L (n = 7,112), and those with unclear diabetes status during follow-up (n = 6,630). This yielded 211,833 eligible participants.

**Figure 1:**
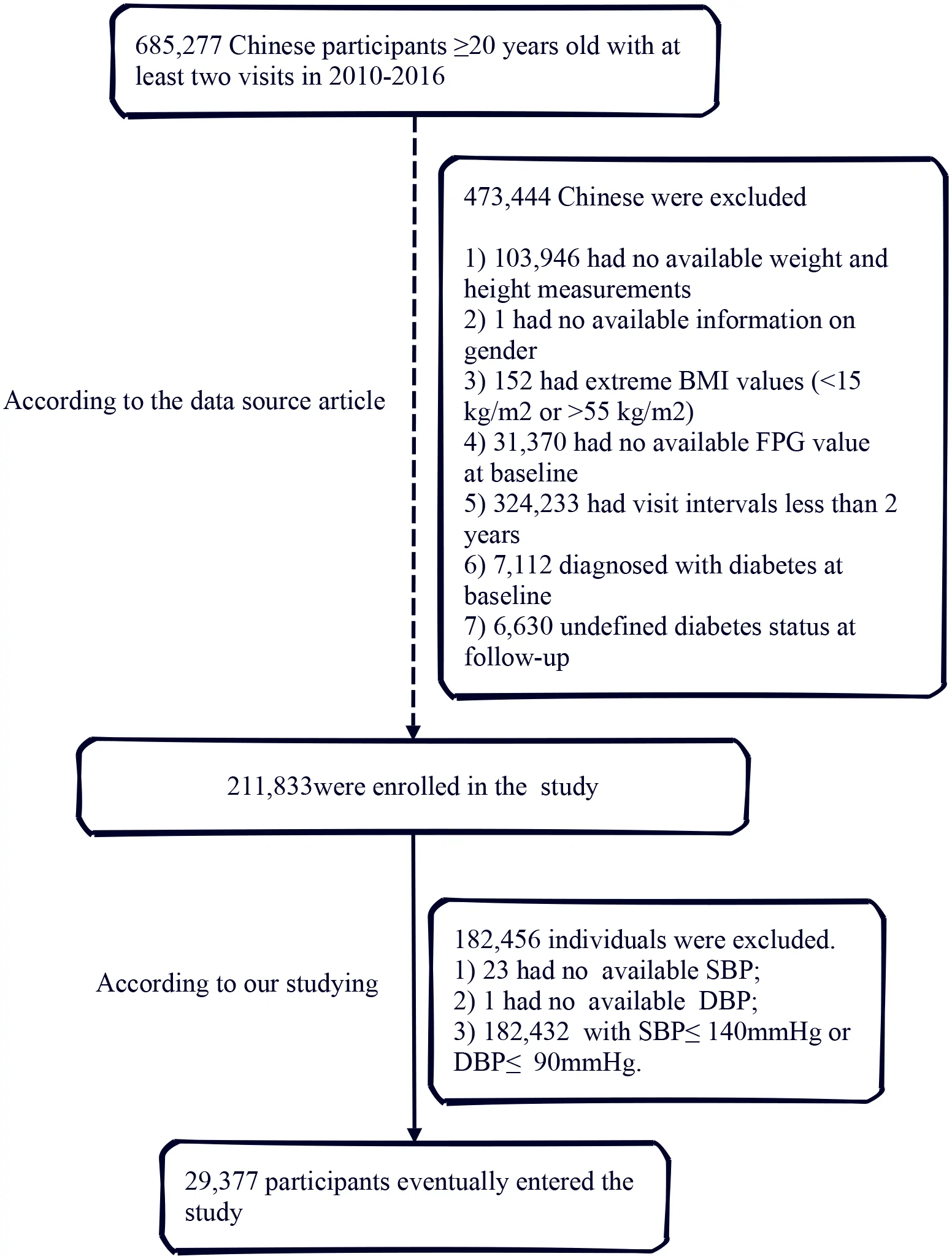
Flowchart of the study participants in the Chinese population.

For the present analysis, we restricted the study population to individuals with elevated blood pressure at baseline, defined as SBP ≥140 mmHg or DBP ≥90 mmHg. After excluding participants with missing BP measurements, a total of 29,377 individuals were included in the final analysis. The study flowchart is shown in **Figure 1**.

### 2.2 Ethical approval

This study was conducted in accordance with the Declaration of Helsinki. The data were derived from the Rich Healthcare Group database, a de-identified health examination database. Ethical approval for the use of this database was obtained from the Rich Healthcare Group Review Board. The Review Board approved the use of the database for retrospective studies and waived the requirement for informed consent because the study involved the use of preexisting de-identified data. As this was a secondary analysis of an existing de-identified database, no additional ethical approval was required.

### 2.3 Data collection and definitions

At baseline, all participants underwent standardized clinical examinations, including anthropometric measurements, blood pressure assessment, and laboratory testing after an overnight fast of at least 8 hours.

Height and weight were measured by trained staff using calibrated instruments, and BMI was calculated as weight (kg) divided by height squared (m²). Blood pressure was measured using a standard mercury sphygmomanometer or automated device after at least 5 minutes of rest in a seated position, with the arm supported at heart level.

In addition to SBP and DBP, the following derived BP parameters were calculated: Mean arterial pressure (MAP): DBP + 1/3 × (SBP − DBP); Pulse pressure (PP): SBP – DBP; Pulse pressure index (PPI): PP/SBP; SBP/DBP ratio: SBP divided by DBP.

Venous blood samples were collected after overnight fasting. FPG was measured using the hexokinase or glucose oxidase method. Total cholesterol (TC), triglycerides (TG), high-density lipoprotein cholesterol (HDL-C), low-density lipoprotein cholesterol (LDL-C), alanine aminotransferase (ALT), blood urea nitrogen (BUN), and serum creatinine (Scr) were measured using standard automated analyzers.

Baseline demographic and lifestyle information, including age, sex, smoking status, drinking status, and family history of diabetes, were collected using standardized questionnaires. Smoking and drinking status were categorized as current, former, or never. A family history of diabetes was defined as having at least one first-degree relative with diabetes.

### 2.4 Follow-up procedures

In this database, participants were routinely encouraged to undergo annual health examinations. The average interval between consecutive visits was approximately 1 year, a frequency consistent with the standard practice for health check-up programs in China. For each follow-up visit, all participants underwent standardized assessments including BP measurement, anthropometric measurements, and laboratory testing after an overnight fast, following the same protocols as at baseline. Information on lifestyle factors was updated using standardized questionnaires at each visit. We included participants who had at least two health examinations during follow-up to ensure ascertainment of incident outcomes. All participants were followed from baseline (first health examination) to the date of the last health examination, the date of diabetes diagnosis, or December 31, 2016, whichever occurred first.

### 2.5 Outcome ascertainment

The primary outcome was incident diabetes during follow-up. Diabetes was defined according to the American Diabetes Association criteria as any of the following: (1) fasting plasma glucose ≥7.0 mmol/L at follow-up visits, (2) self-reported physician-diagnosed diabetes, or (3) use of glucose-lowering medications.

The date of incident diabetes was defined as the first follow-up visit at which any of these criteria were met. Participants who did not develop diabetes were censored at the date of their last follow-up visit. Follow-up time was calculated from baseline to the date of diabetes onset or last follow-up, whichever occurred first.

### 2.6 Statistical analysis

Baseline characteristics were summarized according to incident diabetes status. Continuous variables were presented as mean ± standard deviation (SD) or median (interquartile range, IQR), depending on their distribution, and compared using the Student’s t-test or Mann–Whitney U test as appropriate. Categorical variables were expressed as numbers (percentages) and compared using the chi-square test or Fisher’s exact test. In this study, we employed Multiple Imputation (MI) techniques to handle missing data.

Cox proportional hazards regression models were used to evaluate the associations between BP parameters and incident diabetes. BP parameters were analyzed both as continuous variables and as quartiles, with the lowest quartile serving as the reference group. Hazard ratios (HRs) and 95% confidence intervals (CIs) were estimated.

To assess multicollinearity, variance inflation factors (VIFs) were calculated, and all variables showed VIF <5, indicating no significant collinearity (**Supplementary Table 1**). Three models were constructed: Model 1: unadjusted; Model 2: adjusted for age, sex, and BMI; Model 3: further adjusted for FPG, TC, TG, ALT, BUN, smoking status, drinking status, and family history of diabetes. For continuous analyses, HRs were reported per 10 mmHg increase for SBP, DBP, MAP, and PP, and per unit increase for PPI and the SBP/DBP ratio.

Restricted cubic spline models with three knots (at the 10th, 50th, and 90th percentiles) were used to explore potential non-linear dose–response relationships. Non-linearity was assessed using likelihood ratio tests comparing models with and without spline terms. When significant non-linearity was detected, two-piecewise linear regression models were applied to identify potential threshold effects.

To evaluate the predictive performance of BP parameters, time-dependent receiver operating characteristic (ROC) curve analysis was performed, and the area under the curve (AUC) was calculated. Sensitivity analyses included complete-case analyses, analyses defining incident diabetes solely by FPG ≥7.0 mmol/L, and a fully adjusted analysis restricted to participants with baseline FPG ≤6.1 mmol/L (n = 27,094) to reduce potential reverse causation from baseline hyperglycemia. All statistical analyses were performed using R (R Foundation for Statistical Computing), Free Statistics software (version 2.3), and EmpowerStats (X&Y Solutions, Boston, MA). A two-sided P value <0.05 was considered statistically significant.

## 3 Results

### 3.1 Baseline characteristics

A total of 29,377 participants with elevated blood pressure were included in the final analysis. The mean age was 51.53 ± 14.84 years, and 69.48% were men. During a median follow-up of 2.98 years (interquartile range: 2.14–3.93 years), 1,550 participants (5.28%) developed incident diabetes.

Baseline characteristics stratified by diabetes status are presented in **Table 1**. Compared with participants who did not develop diabetes, those who developed diabetes were older (58.26 ± 13.35 vs. 51.15 ± 14.83 years), had higher BMI (26.72 ± 3.52 vs. 25.21 ± 3.39 kg/m²), and higher fasting plasma glucose levels (5.96 ± 0.70 vs. 5.10 ± 0.63 mmol/L) (all *P* < 0.001).

**Table 1:** Participant characteristics.

Characteristics	Total (n=29,377)	Non-diabetes (n=27,827)	Diabetes (n=1,550)	P-value
Age, years	51.53 ± 14.84	51.15 ± 14.83	58.26 ± 13.35	<0.001
BMI, kg/m2	25.29 ± 3.41	25.21 ± 3.39	26.72 ± 3.52	<0.001
Gender, n (%)				0.190
Male	20410 (69.48%)	19310 (69.39%)	1100 (70.97%)	
Female	8967 (30.52%)	8517 (30.61%)	450 (29.03%)	
BP parameters				
SBP, mmHg	145.42 ± 13.52	145.18 ± 13.44	149.57 ± 14.16	<0.001
DBP, mmHg	89.86 ± 10.20	89.88 ± 10.11	89.59 ± 11.77	0.283
MAP, mmHg	108.38 ± 8.32	108.31 ± 8.24	109.58 ± 9.59	<0.001
PP, mmHg	55.55 ± 16.57	55.31 ± 16.49	59.97 ± 17.38	<0.001
PPI	0.38 ± 0.09	0.38 ± 0.09	0.40 ± 0.09	<0.001
SBP/DBP	1.64 ± 0.25	1.64 ± 0.25	1.70 ± 0.27	<0.001
FPG, mmol/L	5.15 ± 0.66	5.10 ± 0.63	5.96 ± 0.70	<0.001
TC, mmol/L	5.00 ± 0.94	4.99 ± 0.94	5.13 ± 0.94	<0.001
TG, mmol/L	1.78 ± 1.32	1.75 ± 1.30	2.21 ± 1.62	<0.001
HDL-C, mmol/L	1.40 ± 0.32	1.41 ± 0.32	1.36 ± 0.31	<0.001
LDL-C, mmol/L	3.64 ± 1.19	3.65 ± 1.19	3.57 ± 1.19	0.012
ALT, U/L	22.60 (16.00-34.00)	22.30 (16.00-34.00)	26.20 (18.00-40.00)	<0.001
BUN, mmol/L	5.09 ± 1.46	5.09 ± 1.45	5.21 ± 1.55	0.001
Scr, μmol/L	74.35 ± 17.82	74.40 ± 17.85	73.51 ± 17.34	0.056
Drinking status, n (%)				0.043
Current drinker	1151 (3.92%)	1086 (3.90%)	65 (4.19%)	
Ever drinker	4189 (14.26%)	3936 (14.14%)	253 (16.32%)	
Never drinker	24037 (81.82%)	22805 (81.95%)	1232 (79.48%)	
Smoking status, n (%)				<0.001
Current smoker	6902 (23.49%)	6453 (23.19%)	449 (28.97%)	
Ever smoker	1250 (4.26%)	1137 (4.09%)	113 (7.29%)	
Never smoker	21225 (72.25%)	20237 (72.72%)	988 (63.74%)	
Family history of diabetes, n (%)				<0.001
No	28934 (98.49%)	27424 (98.55%)	1510 (97.42%)	
Yes	443 (1.51%)	403 (1.45%)	40 (2.58%)	
Follow-up (years)	2.98 (2.14-3.93)	2.98 (2.13-3.92)	3.18 (2.57-4.01)	<0.001

Values are n (%), means or medians (quartiles).

BMI, body-mass index; FPG, fasting plasma glucose; SBP, systolic blood pressure; DBP, diastolic blood pressure; TC, total cholesterol; TG, triglycerides; HDL-C, high-density lipoprotein cholesterol; LDL-C, low-density lipoprotein cholesterol; ALT, alanine aminotransferase; BUN, blood urea nitrogen; Scr, serum creatinine; MAP, mean arterial pressure; PP, Pulse Pressure; PPI, pulse pressure index.

Regarding blood pressure parameters, participants who developed diabetes had significantly higher SBP, MAP, PP, PPI, and SBP/DBP ratio (all *P* < 0.001), whereas DBP did not differ significantly between groups (*P* = 0.283). In addition, the diabetes group exhibited a less favorable metabolic profile, including higher levels of total cholesterol, triglycerides, alanine aminotransferase, and blood urea nitrogen, as well as lower HDL-C levels. The distribution of blood pressure parameters in the entire cohort is shown in **Supplementary Figure 1**. As shown in **Supplementary Figure 2**, incidence of diabetes by sex and age group (<30, 30-39, 40-49, 50-59, 60-69, ≥70 years) shown as percentages.

Across quartiles of BP parameters (**Supplementary Tables 2-7**), higher SBP, PP, and related indices were generally associated with older age and worse metabolic profiles, suggesting a clustering of cardiometabolic risk factors.

### 3.2 Associations between blood pressure parameters and incident diabetes

In univariate Cox regression analyses (**Supplementary Table 8**), SBP, MAP, PP, PPI, and the SBP/DBP ratio were all significantly associated with incident diabetes, with SBP showing the strongest association (per 10 mmHg increase: HR 1.27, 95% CI 1.23–1.31, *P* < 0.001).

After multivariable adjustment (Model 3), SBP remained significantly associated with incident diabetes, with each 10-mmHg increase associated with a 9% higher risk (HR 1.09, 95% CI 1.05–1.13, *P* < 0.001). Similarly, MAP (HR 1.07, 95% CI 1.02–1.13, *P* = 0.009) and PP (HR 1.06, 95% CI 1.03–1.10, P <0.001) were independently associated with increased diabetes risk. PPI (HR 2.47, 95% CI 1.36–4.46, *P* = 0.003) and the SBP/DBP ratio (HR 1.38, 95% CI 1.13–1.70, *P* = 0.002) also showed significant associations. In contrast, DBP was not significantly associated with incident diabetes after full adjustment (HR 1.01, 95% CI 0.96–1.06, *P* = 0.737) (**Table 2**).

**Table 2:** Associations between BP parameters and the incident diabetes in different model.

Exposure	Model I (HR,95%CI) P	Model II (HR,95%CI) P	Model III (HR,95%CI) P
SBP (10 mmHg)	1.27 (1.23, 1.31) <0.0001	1.13 (1.09, 1.17) <0.0001	1.09 (1.05, 1.13) <0.0001
Q1	Ref	Ref	Ref
Q2	1.61 (1.36, 1.90) <0.0001	1.41 (1.19, 1.67) <0.0001	1.29 (1.08, 1.52) 0.0037
Q3	1.90 (1.62, 2.23) <0.0001	1.49 (1.26, 1.75) <0.0001	1.34 (1.14, 1.58) 0.0005
Q4	2.62 (2.25, 3.05) <0.0001	1.68 (1.43, 1.97) <0.0001	1.45 (1.24, 1.70) <0.0001
DBP (10 mmHg)	0.93 (0.89, 0.98) 0.0049	0.96 (0.91, 1.01) 0.0802	1.01 (0.96, 1.06) 0.7368
Q1	Ref	Ref	Ref
Q2	0.74 (0.65, 0.85) <0.0001	0.83 (0.72, 0.95) 0.0079	0.92 (0.79, 1.06) 0.2251
Q3	0.59 (0.51, 0.69) <0.0001	0.71 (0.61, 0.83) <0.0001	0.86 (0.74, 1.01) 0.0694
Q4	0.86 (0.76, 0.98) 0.0229	0.92 (0.80, 1.05) 0.2064	1.02 (0.89, 1.17) 0.7771
MAP (10 mmHg)	1.17 (1.11, 1.24) <0.0001	1.06 (1.00, 1.12) 0.0472	1.07 (1.02, 1.13) 0.0094
Q1	Ref	Ref	Ref
Q2	0.86 (0.74, 1.00) 0.0571	0.88 (0.76, 1.03) 0.1196	0.94 (0.81, 1.10) 0.4546
Q3	1.01 (0.87, 1.16) 0.9362	0.93 (0.81, 1.07) 0.3252	1.04 (0.90, 1.20) 0.6034
Q4	1.35 (1.18, 1.55) <0.0001	1.08 (0.94, 1.24) 0.2642	1.14 (0.99, 1.31) 0.0634
PP (10 mmHg)	1.22 (1.19, 1.26) <0.0001	1.12 (1.08, 1.15) <0.0001	1.06 (1.03, 1.10) 0.0002
Q1	Ref	Ref	Ref
Q2	1.52 (1.29, 1.78) <0.0001	1.31 (1.11, 1.53) 0.0011	1.30 (1.11, 1.53) 0.0012
Q3	1.65 (1.42, 1.93) <0.0001	1.38 (1.18, 1.61) <0.0001	1.25 (1.07, 1.46) 0.0048
Q4	2.40 (2.08, 2.78) <0.0001	1.62 (1.38, 1.89) <0.0001	1.32 (1.13, 1.55) 0.0005
PPI	25.47 (14.65, 44.26) <0.0001	6.37 (3.51, 11.57) <0.0001	2.47 (1.36, 4.46) 0.0028
Q1	Ref	Ref	Ref
Q2	1.41 (1.20, 1.65) <0.0001	1.24 (1.06, 1.45) 0.0076	1.23 (1.05, 1.44) 0.0105
Q3	1.53 (1.31, 1.79) <0.0001	1.28 (1.09, 1.50) 0.0021	1.13 (0.96, 1.32) 0.1371
Q4	2.14 (1.85, 2.47) <0.0001	1.55 (1.33, 1.81) <0.0001	1.29 (1.11, 1.50) 0.0012
SBP/DBP	3.06 (2.55, 3.68) <0.0001	1.93 (1.58, 2.36) <0.0001	1.38 (1.13, 1.70) 0.0019
Q1	Ref	Ref	Ref
Q2	1.43 (1.23, 1.67) <0.0001	1.26 (1.08, 1.46) 0.0036	1.25 (1.07, 1.46) 0.0042
Q3	1.50 (1.29, 1.75) <0.0001	1.27 (1.08, 1.48) 0.0028	1.12 (0.96, 1.31) 0.1535
Q4	2.15 (1.87, 2.49) <0.0001	1.53 (1.31, 1.78) <0.0001	1.26 (1.08, 1.47) 0.0028

Model I: we did not adjust other covariates.

Model II: we adjust age, gender and BMI.

Model III: we adjust variables in Adjust II+ FPG, TC, TG, ALT, BUN, smoking status, Drinking status, Family history of diabetes. HR, Hazard Ratio; CI, Confidence Interval.

In quartile analyses, SBP demonstrated a clear dose–response relationship with diabetes risk. Compared with the lowest quartile, the fully adjusted HRs across increasing quartiles were 1.29 (95% CI 1.08–1.52), 1.34 (95% CI 1.14–1.58), and 1.45 (95% CI 1.24–1.70), respectively (*P* for trend <0.001). A similar graded association was observed for PP, whereas the association for DBP was attenuated and no longer significant after adjustment (**Table 2**).

### 3.3 Non-linear relationships between BP parameters and diabetes risk

**Figure 2:**
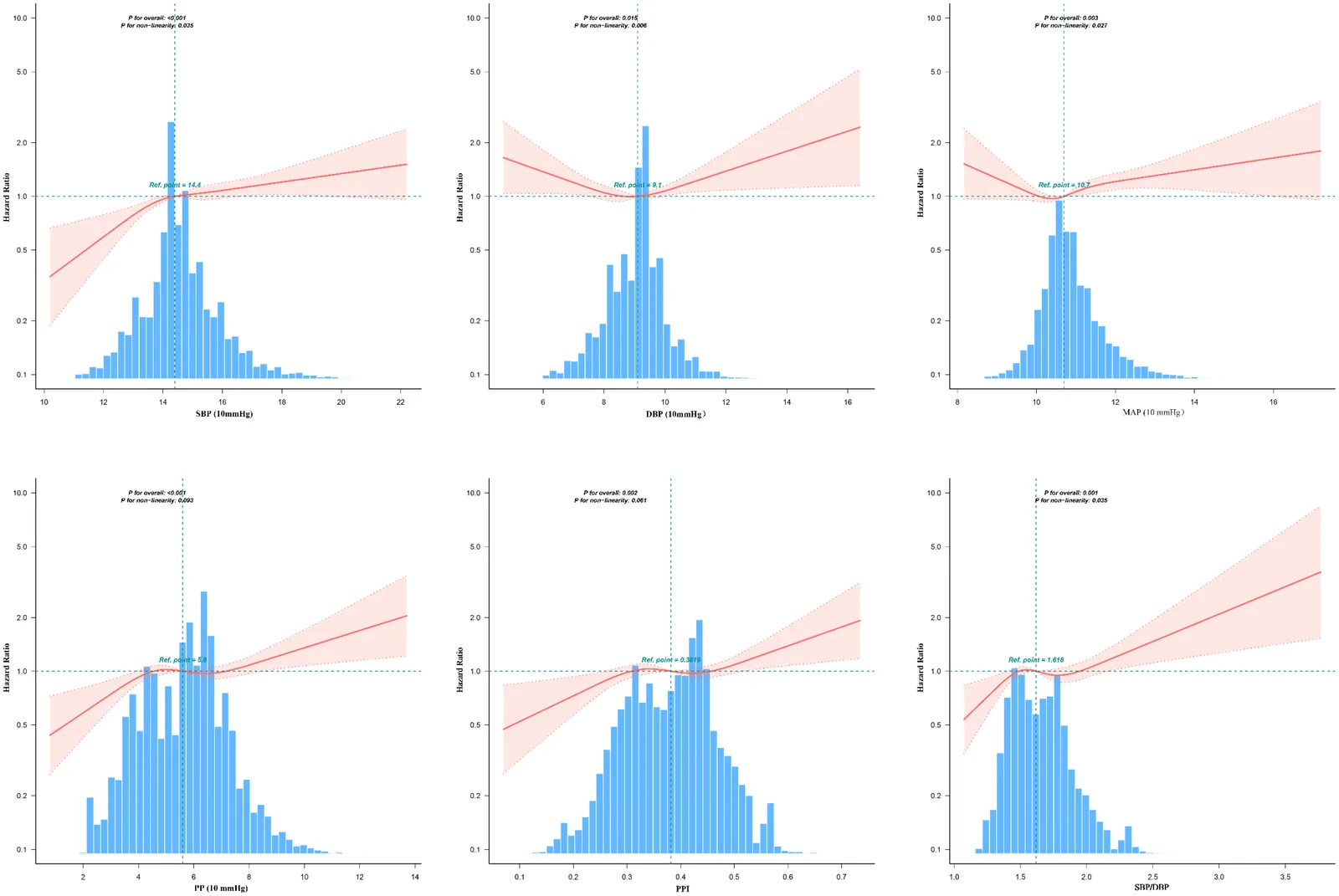
Non-linear relationships between BP Parameters and diabetes risk. Restricted cubic spline plots illustrate the dose–response association of each BP Parameters with incident diabetes. The solid red line represents the estimated HR, and the red dashed shading indicates the 95% confidence interval. A: SBP; B: DBP; C: MAP; D: PP; E: PPI; F: SBP/DBP. P-values for non-linearity were <0.001 for all indices.

Restricted cubic spline analyses revealed significant non-linear associations between several BP parameters and incident diabetes (**Figure 2**). Significant non-linearity was observed for SBP, DBP, MAP, and the SBP/DBP ratio (P for non-linearity <0.05), whereas PP and PPI showed largely linear relationships.

Threshold effect analyses identified distinct inflection points for several BP parameters (**Table 3**). For SBP, the inflection point was 139 mmHg. Below this threshold, each 10 mmHg increase in SBP was associated with a substantially higher diabetes risk (HR 1.29, 95% CI 1.12–1.49), whereas above this threshold, the association was attenuated (HR 1.05, 95% CI 1.01–1.10).

**Table 3:** Nonlinear associations between BP Parameters and diabetes risk.

IR indices	SBP (10 mmHg)	DBP (10 mmHg)	MAP (10 mmHg)	PP (10 mmHg)	PPI	SBP/DBP
Model I	1.09 (1.05, 1.13) <0.0001	1.01 (0.96, 1.06) 0.7368	1.07 (1.02, 1.13) 0.0094	1.06 (1.03, 1.10) 0.0002	2.47 (1.36, 4.46) 0.0028	1.38 (1.13, 1.70) 0.0019
Model II						
Inflection points	13.9	8.4	10.27	4.4	0.51	1.45
<K	1.29 (1.12, 1.49) 0.0004	0.85 (0.76, 0.96) 0.0067	0.80 (0.65, 0.99) 0.0378	1.19 (1.04, 1.36) 0.0118	2.04 (1.05, 3.94) 0.0347	3.45 (1.00, 11.87) 0.0492
≥K	1.05 (1.01, 1.10) 0.0221	1.09 (1.02, 1.16) 0.0137	1.13 (1.06, 1.20) 0.0002	1.04 (1.00, 1.08) 0.0848	26.66 (0.69, 1037.56) 0.0788	1.25 (0.98, 1.60) 0.0756
P for log-likelihood ratio test	0.009	0.006	0.003	0.083	0.205	0.013

Data were presented as HR (95% CI) P value; Model I, linear analysis; Model II, non-linear analysis. Adjusted for age, gender and BMI, FPG, TC, TG, ALT, BUN, smoking status, Drinking status, Family history of diabetes. HR Hazard Ratio, CI Confidence Interval. K indicates inflection point. P for log-likelihood ratio test < 0.05 indicates that model II is significantly different from Model I.

For DBP, a threshold was identified at 84 mmHg, with an inverse association below the threshold (HR 0.85, 95% CI 0.76–0.96) and a positive association above it (HR 1.09, 95% CI 1.02–1.16), suggesting a J-shaped relationship. Similarly, MAP showed a threshold at 102.7 mmHg, with a negative association below and a positive association above the threshold. The SBP/DBP ratio also demonstrated a non-linear pattern, with a stronger association below a threshold of 1.45.

### 3.4 Predictive performance of BP parameters

The predictive performance of BP parameters for incident diabetes was evaluated using time-dependent ROC curve analysis (**Supplementary Table 9**). Among all parameters, SBP demonstrated the highest discriminative ability (AUC 0.5875, 95% CI 0.5730–0.6020), although the overall performance was modest.

Other parameters, including PP, PPI, and the SBP/DBP ratio, showed slightly lower AUC values (approximately 0.56–0.57), whereas MAP and especially DBP exhibited limited predictive value (DBP AUC 0.5077), indicating near-random discrimination.

### 3.5 Sensitivity analyses

Sensitivity analyses confirmed the robustness of the main findings. Analyses restricted to complete cases yielded similar results (**Supplementary Table 10**). In addition, when incident diabetes was defined solely based on FPG ≥7.0 mmol/L, excluding self-reported cases, the associations between BP parameters and diabetes risk remained largely unchanged (**Supplementary Table 11**).

## 4 Discussion

In this large prospective cohort of Chinese adults with elevated blood pressure, we comprehensively evaluated the associations between multiple BP parameters and incident diabetes. Several key findings emerged. First, SBP, MAP, PP, PPI, and the SBP/DBP ratio were independently associated with an increased risk of diabetes, whereas DBP was not. Second, significant non-linear relationships were observed for SBP, DBP, MAP, and the SBP/DBP ratio, with distinct threshold effects. Third, among all parameters, SBP demonstrated the best predictive performance for incident diabetes.

The positive association between SBP and incident diabetes observed in our study is consistent with previous large-scale epidemiological studies. A meta-analysis by Emdin et al. reported that each 20 mmHg increase in SBP was associated with a 77% higher risk of diabetes, and similar associations have been observed in long-term cohort studies such as the Atherosclerosis Risk in Communities (ARIC) study (5, 20). Recent evidence further suggests that the relationship between blood pressure and metabolic outcomes may follow a graded, and potentially non-linear, pattern across a wide range of blood pressure levels (21). Importantly, most previous studies have examined hypertension as a binary exposure in general populations, whereas relatively few have evaluated the differential roles of multiple blood pressure parameters within individuals with elevated blood pressure. By specifically focusing on this high-risk population, our study extends the existing evidence and provides a more refined assessment of blood pressure–related metabolic risk. Notably, the stronger association observed below the threshold suggests that even modest elevations in SBP may contribute to diabetes development, highlighting the potential importance of early intervention before overt hypertension is established.

In contrast, DBP was not independently associated with diabetes risk after multivariable adjustment in our study. Previous evidence suggests that although both SBP and DBP may be associated with diabetes risk, SBP generally shows stronger and more consistent predictive value (22). This difference may be explained by their distinct physiological trajectories, as SBP increases with age and reflects arterial stiffness, whereas DBP tends to plateau or decline in later life (23). We also found that PP was independently associated with incident diabetes, supporting the role of arterial stiffness in diabetes pathogenesis. Prospective studies using cfPWV have shown that arterial stiffness is associated with an increased risk of diabetes (24). This association may reflect early vascular dysfunction preceding overt hyperglycemia, as endothelial and vascular impairment have been shown to occur in the early stages of metabolic dysfunction (25).

A key finding of this study is the identification of non-linear relationships between blood pressure parameters and diabetes risk. Notably, SBP exhibited a threshold effect at approximately 139 mmHg, with a stronger association observed below this level. This threshold is close to the diagnostic cut-off for hypertension in current guidelines (26), suggesting potential clinical relevance. The attenuation of risk above this threshold may reflect several factors. One possible explanation is the increased use of antihypertensive medications among individuals with higher BP levels, which may modify diabetes risk. Previous studies have shown that different classes of antihypertensive agents have heterogeneous metabolic effects, with renin–angiotensin system inhibitors potentially reducing diabetes risk, whereas β-blockers and thiazide diuretics may increase it (19). In addition, DBP and MAP demonstrated J-shaped relationships with diabetes risk, characterized by inverse associations at lower levels and positive associations above specific thresholds. While J-shaped associations between blood pressure and cardiovascular and cerebrovascular outcomes have been well documented, evidence regarding such non-linear patterns in relation to diabetes remains limited (27, 28). Nevertheless, recent studies applying spline-based models have highlighted the importance of considering non-linear relationships between BP and metabolic outcomes, supporting the robustness of our findings (29).

Several biological mechanisms may underlie the observed associations. First, elevated BP is closely linked to arterial stiffness and endothelial dysfunction, which have been shown to impair insulin-mediated glucose uptake in peripheral tissues (25). Second, activation of the renin–angiotensin–aldosterone system (RAAS), a hallmark of hypertension, has been implicated in the development of insulin resistance. Angiotensin II can interfere with insulin signaling pathways and promote oxidative stress and inflammation (30, 31). Third, elevated BP frequently coexists with other metabolic abnormalities, including obesity, dyslipidemia, and chronic low-grade inflammation, which collectively contribute to insulin resistance and β-cell dysfunction (32, 33). Although we adjusted for these factors, the associations remained significant, suggesting an independent contribution of BP parameters. Finally, the observed non-linear relationships may reflect complex interactions between hemodynamic factors and tissue perfusion. At very low BP levels, reduced microvascular perfusion may impair glucose delivery and insulin action, potentially contributing to insulin resistance (34, 35).

Our restricted cubic spline analyses revealed non-linear relationships with threshold effects. These findings warrant careful interpretation. The stronger SBP–diabetes association below 139 mmHg indicates that incremental BP elevations within the high-normal range may have proportionally greater metabolic impact, rather than suggesting that lower BP is harmful. The J-shaped patterns for DBP and MAP likely reflect reverse causation or unmeasured confounding rather than true causal effects, consistent with a recent large-scale meta-analysis demonstrating that J-shaped associations were attenuated after excluding populations with baseline diseases (21). Therefore, these thresholds (SBP ~139 mmHg, DBP ~84 mmHg, MAP ~102.7 mmHg) should be viewed as reference points for risk identification, not as treatment targets.

This study has several strengths. The large sample size and substantial number of incident diabetes cases ensured adequate statistical power, while the comprehensive assessment of multiple blood pressure parameters enabled direct comparison of their predictive value. In addition, the application of restricted cubic spline models allowed a flexible evaluation of potential non-linear relationships. Importantly, the focus on individuals with elevated BP further enhances the clinical relevance of our findings. These findings also have important clinical implications. First, SBP may serve as a simple and practical marker for diabetes risk stratification in individuals with elevated BP. Given that BP measurement is routinely performed in clinical practice, incorporating SBP into risk assessment requires no additional cost. Second, the identified thresholds (SBP ~139 mmHg, DBP ~84 mmHg, MAP ~102.7 mmHg) may provide additional reference points for identifying individuals at high risk of diabetes. While current guidelines primarily focus on cardiovascular outcomes, our findings suggest that glucometabolic risk, in addition to cardiovascular risk, should be considered in BP management (26). Third, the independent associations of PP and the SBP/DBP ratio highlight the potential value of incorporating arterial stiffness-related parameters into routine clinical assessment, particularly in older individuals.

Several limitations should be acknowledged. First, BP was measured at baseline and subsequent health examinations, but longitudinal BP trajectories and time-varying changes in antihypertensive treatment could not be modeled. Second, diabetes ascertainment did not include oral glucose tolerance testing or glycated hemoglobin, so some cases may have been missed. Third, information on antihypertensive medication use was unavailable, creating an important source of residual confounding; class-specific metabolic effects may have influenced both the estimated associations and the shapes of the non-linear relationships. Fourth, the cohort comprised Chinese adults who voluntarily attended health examinations, which may introduce selection bias and limit generalizability to other ethnic groups and populations without regular health screening. Fifth, the median follow-up of approximately 3 years limits inference about long-term risk. Finally, the observational design precludes causal conclusions, and reverse causation cannot be completely excluded despite the sensitivity analyses.

Future studies should evaluate these findings in prospective cohorts with repeated and ambulatory BP measurements, detailed antihypertensive medication records, and diabetes ascertainment incorporating FPG, glycated hemoglobin, and oral glucose tolerance testing. External validation in other ethnic groups and in individuals with normal BP is also needed. Prediction models should assess whether BP parameters provide incremental value beyond established diabetes risk factors using appropriate measures of calibration and discrimination. Mechanistic studies may clarify the roles of arterial stiffness, inflammation, oxidative stress, and endothelial dysfunction. Randomized trials could compare the effects of antihypertensive drug classes and BP-lowering strategies on diabetes incidence; however, the data-derived inflection points identified here should not be used as intervention targets unless prospectively validated.

## 5 Conclusion

In this large cohort of Chinese adults with elevated BP, five of the six BP parameters examined—SBP, MAP, PP, PPI, and the SBP/DBP ratio—were independently associated with incident diabetes, whereas DBP was not. Significant non-linear relationships were observed for SBP, DBP, MAP, and the SBP/DBP ratio. SBP had the highest predictive performance, but its modest discrimination limits its use as a stand-alone predictor. BP parameters may therefore serve as readily available supplementary indicators for diabetes risk stratification in this high-risk population.

## Data Availability

The original contributions presented in the study are included in the article/**Supplementary Material**. Further inquiries can be directed to the corresponding authors.
