# Supplementary material for: Multiple blood pressure parameters and risk of incident diabetes in adults with elevated blood pressure: a cohort study with non-linear analyses
Source: Front Endocrinol (Lausanne). 2026 Aug 27;17:1887291. doi: 10.3389/fendo.2026.1887291 (PMC13558090; doi:10.3389/fendo.2026.1887291)
Supplement: Supplementary file 1 [file Supplementaryfile1.docx]

**Multiple blood pressure parameters and risk of incident diabetes in adults with elevated blood pressure: a cohort study with non-linear analyses**

Qiaofeng Wu^a,*^, Yuting Gao^b,*^, Anwen Yu^a,*^, Zhenhua Huang^c,#^, Xin Li^a,#^

^a^ Department of Emergency Medicine , Guangdong Provincial People's Hospital, Guangdong Academy of Medical Sciences, Southern Medical University

^b^ Department of Endocrinology and Metabolism, Peking University Third Hospital

^c^ Department of Emergency Medicine, the First Affiliated Hospital of Shenzhen University, Shenzhen Second People’s Hospital

***Qiaofeng Wu, Yuting Gao, Anwen Yu** contributed equally to this workand share first authorship.

^#^Corresponding authors:

**Zhenhua Huang**

Department of Emergency Medicine, the First Affiliated Hospital of Shenzhen University, Shenzhen Second People’s Hospital. Email: [*huangzhh12306@163.com.*](mailto:huangzhh12306@163.com.)

**Xin Li**

Department of Emergency Medicine, Guangdong Provincial People' s Hospital (Guangdong Academy of Medical Sciences), Southern Medical University, Guangzhou, 518001, China Email:  [*sylixin@scut.edu.cn*](mailto:yuanqinghua@sysush.com)

**Table S1. Variance inflation factors for multicollinearity assessment in the initial model.**

| Variable | VIF Value | |
| --- | --- | --- |
|  | **Step1** | **Step2** |
| Age (years) | 1.4 | 1.4 |
| Gender | 1.9 | 1.9 |
| BMI (kg/m2) | 1.3 | 1.3 |
| FPG (mmol/L) | 1.1 | 1.1 |
| TC (mmol/L) | 1.6 | 1.6 |
| TG (mmol/L) | 1.3 | 1.3 |
| HDL-c (mmol/L) | 1.2 | 1.2 |
| LDL-c (mmol/L) | 1.4 | 1.4 |
| ALT (U/L) | 1.3 | 1.3 |
| BUN (mmol/L) | 1.2 | 1.2 |
| Scr (μmol/L) | 1.6 | 1.6 |
| Smoking Status | 1.2 | 1.2 |
| Drinking Status | 1.1 | 1.1 |
| Family History of diabetes | 1 | 1 |

**Abbreviations**: BMI, body-mass index; FPG, fasting plasma glucose; SBP, systolic blood pressure; DBP, diastolic blood pressure; TC, total cholesterol; TG, triglycerides; HDL-C, high-density lipoprotein cholesterol; LDL-C, low-density lipoprotein cholesterol; ALT, alanine aminotransferase; BUN, blood urea nitrogen; Scr, serum creatinine; MAP, mean arterial pressure; PP, Pulse Pressure; PPI, pulse pressure index.

**Note**: Variance inflation factor (VIF) was calculated as VIF = 1/(1-R²), where R² was obtained from the regression of each variable on all other variables. Variables are arranged in descending order of VIF values. A VIF ≥ 5 was considered to indicate significant multicollinearity. In this initial assessment, all variables showed VIF values < 5, suggesting no significant multicollinearity among the variables.

**Table S2. Participant characteristics across SBP quartiles**

| Characteristics | Q1(n=6959) | Q2 (n=6856) | Q3 (n=7626) | Q4 (n=7936) | P-value |
| --- | --- | --- | --- | --- | --- |
| Age, years | 46.17 ± 11.69 | 49.28 ± 14.43 | 52.21 ± 15.25 | 57.51 ± 15.03 | <0.001 |
| BMI, kg/m2 | 25.04 ± 3.41 | 25.16 ± 3.33 | 25.30 ± 3.42 | 25.60 ± 3.46 | <0.001 |
| FPG, mmol/L | 5.02 ± 0.67 | 5.12 ± 0.65 | 5.18 ± 0.64 | 5.25 ± 0.67 | <0.001 |
| TC, mmol/L | 4.92 ± 0.90 | 4.94 ± 0.93 | 5.01 ± 0.94 | 5.12 ± 0.96 | <0.001 |
| TG, mmol/L | 1.77 ± 1.39 | 1.73 ± 1.24 | 1.75 ± 1.28 | 1.85 ± 1.36 | <0.001 |
| HDL-C, mmol/L | 1.38 ± 0.32 | 1.40 ± 0.32 | 1.41 ± 0.32 | 1.43 ± 0.32 | <0.001 |
| LDL-C, mmol/L | 3.57 ± 1.13 | 3.60 ± 1.16 | 3.63 ± 1.18 | 3.75 ± 1.27 | <0.001 |
| ALT, U/L | 23.40 (16.00-36.00) | 23.00 (16.00-34.90) | 22.20 (15.90-34.00) | 21.65 (16.00-32.00) | <0.001 |
| BUN, mmol/L | 4.97 ± 1.34 | 5.04 ± 1.42 | 5.11 ± 1.48 | 5.22 ± 1.56 | <0.001 |
| Scr, μmol/L | 74.53 ± 15.78 | 74.46 ± 16.01 | 74.32 ± 16.85 | 74.14 ± 21.51 | 0.557 |
| Gender, n (%) |  |  |  |  | <0.001 |
| Male | 5080 (73.00%) | 4959 (72.33%) | 5289 (69.35%) | 5082 (64.04%) |  |
| Female | 1879 (27.00%) | 1897 (27.67%) | 2337 (30.65%) | 2854 (35.96%) |  |
| Smoking Status, n (%) |  |  |  |  | <0.001 |
| Current smoker | 1784 (25.64%) | 1662 (24.24%) | 1705 (22.36%) | 1751 (22.06%) |  |
| Ever smoker | 362 (5.20%) | 294 (4.29%) | 326 (4.27%) | 268 (3.38%) |  |
| Never smoker | 4813 (69.16%) | 4900 (71.47%) | 5595 (73.37%) | 5917 (74.56%) |  |
| Drinking Status, n (%) |  |  |  |  | <0.001 |
| Current drinker | 225 (3.23%) | 267 (3.89%) | 308 (4.04%) | 351 (4.42%) |  |
| Ever drinker | 1239 (17.80%) | 1057 (15.42%) | 1028 (13.48%) | 865 (10.90%) |  |
| Never drinker | 5495 (78.96%) | 5532 (80.69%) | 6290 (82.48%) | 6720 (84.68%) |  |
| Family History of Diabetes, n (%) |  |  |  |  | <0.001 |
| No | 6825 (98.07%) | 6749 (98.44%) | 7508 (98.45%) | 7852 (98.94%) |  |
| Yes | 134 (1.93%) | 107 (1.56%) | 118 (1.55%) | 84 (1.06%) |  |
| Follow-up (years) | 3.02 (2.17-3.99) | 2.98 (2.14-3.93) | 2.97 (2.13-3.90) | 2.96 (2.13-3.88) | <0.001 |
| Diabetes in follow-up, n (%) |  |  |  |  | <0.001 |
| No | 6724 (96.62%) | 6535 (95.32%) | 7212 (94.57%) | 7356 (92.69%) |  |
| Yes | 235 (3.38%) | 321 (4.68%) | 414 (5.43%) | 580 (7.31%) |  |

Values are n (%), means or medians (quartiles)

BMI, body-mass index; FPG, fasting plasma glucose; TC, total cholesterol; TG, triglycerides; HDL-C, high-density lipoprotein cholesterol; LDL-C, low-density lipoprotein cholesterol; ALT, alanine aminotransferase; BUN, blood urea nitrogen; Scr, serum creatinine.

**Table S3. Participant characteristics across DBP quartiles**

| Characteristics | Q1(n=7293) | Q2 (n=7266) | Q3 (n=6555) | Q4 (n=8263) | P-value |
| --- | --- | --- | --- | --- | --- |
| Age, years | 55.29 ± 17.90 | 51.61 ± 14.69 | 48.94 ± 12.93 | 50.19 ± 12.57 | <0.001 |
| BMI, kg/m2 | 24.77 ± 3.39 | 25.19 ± 3.38 | 25.26 ± 3.35 | 25.85 ± 3.44 | <0.001 |
| FPG, mmol/L | 5.22 ± 0.64 | 5.16 ± 0.65 | 5.08 ± 0.67 | 5.12 ± 0.69 | <0.001 |
| TC, mmol/L | 4.98 ± 0.97 | 5.01 ± 0.93 | 4.98 ± 0.92 | 5.04 ± 0.93 | <0.001 |
| TG, mmol/L | 1.61 ± 1.12 | 1.73 ± 1.19 | 1.82 ± 1.43 | 1.93 ± 1.47 | <0.001 |
| HDL-C, mmol/L | 1.42 ± 0.32 | 1.41 ± 0.32 | 1.39 ± 0.32 | 1.39 ± 0.33 | <0.001 |
| LDL-C, mmol/L | 3.61 ± 1.15 | 3.65 ± 1.19 | 3.61 ± 1.17 | 3.69 ± 1.23 | <0.001 |
| ALT, U/L | 20.30 (15.00-30.00) | 22.70 (16.00-34.00) | 23.20 (16.15-35.70) | 24.00 (17.00-36.70) | <0.001 |
| BUN, mmol/L | 5.16 ± 1.51 | 5.11 ± 1.47 | 5.03 ± 1.39 | 5.07 ± 1.45 | <0.001 |
| Scr, μmol/L | 73.21 ± 16.86 | 74.34 ± 20.84 | 74.41 ± 16.01 | 75.33 ± 17.06 | <0.001 |
| Gender, n (%) |  |  |  |  | <0.001 |
| Male | 4484 (61.48%) | 5038 (69.34%) | 4757 (72.57%) | 6131 (74.20%) |  |
| Female | 2809 (38.52%) | 2228 (30.66%) | 1798 (27.43%) | 2132 (25.80%) |  |
| Smoking Status, n (%) |  |  |  |  | <0.001 |
| Current smoker | 1204 (16.51%) | 1669 (22.97%) | 1689 (25.77%) | 2340 (28.32%) |  |
| Ever smoker | 283 (3.88%) | 287 (3.95%) | 293 (4.47%) | 387 (4.68%) |  |
| Never smoker | 5806 (79.61%) | 5310 (73.08%) | 4573 (69.76%) | 5536 (67.00%) |  |
| Drinking Status, n (%) |  |  |  |  | <0.001 |
| Current drinker | 192 (2.63%) | 292 (4.02%) | 256 (3.91%) | 411 (4.97%) |  |
| Ever drinker | 800 (10.97%) | 1038 (14.29%) | 1054 (16.08%) | 1297 (15.70%) |  |
| Never drinker | 6301 (86.40%) | 5936 (81.70%) | 5245 (80.02%) | 6555 (79.33%) |  |
| Family History of Diabetes, n (%) |  |  |  |  | 0.092 |
| No | 7200 (98.72%) | 7157 (98.50%) | 6437 (98.20%) | 8140 (98.51%) |  |
| Yes | 93 (1.28%) | 109 (1.50%) | 118 (1.80%) | 123 (1.49%) |  |
| Follow-up (years) | 2.97 (2.12-3.88) | 2.97 (2.14-3.92) | 2.99 (2.15-3.95) | 3.00 (2.16-3.95) | <0.001 |
| Diabetes in follow-up, n (%) |  |  |  |  | <0.001 |
| No | 6845 (93.86%) | 6920 (95.24%) | 6288 (95.93%) | 7774 (94.08%) |  |
| Yes | 448 (6.14%) | 346 (4.76%) | 267 (4.07%) | 489 (5.92%) |  |

Values are n (%), means or medians (quartiles)

BMI, body-mass index; FPG, fasting plasma glucose; TC, total cholesterol; TG, triglycerides; HDL-C, high-density lipoprotein cholesterol; LDL-C, low-density lipoprotein cholesterol; ALT, alanine aminotransferase; BUN, blood urea nitrogen; Scr, serum creatinine.

**Table S4. Participant characteristics across MAP quartiles**

| Characteristics | Q1(n=7107) | Q2 (n=7017) | Q3 (n=7800) | Q4 (n=7453) | P-value |
| --- | --- | --- | --- | --- | --- |
| Age, years | 51.54 ± 17.24 | 49.55 ± 14.15 | 51.37 ± 13.88 | 53.54 ± 13.66 | <0.001 |
| BMI, kg/m2 | 24.64 ± 3.40 | 25.11 ± 3.36 | 25.45 ± 3.32 | 25.90 ± 3.45 | <0.001 |
| FPG, mmol/L | 5.15 ± 0.65 | 5.11 ± 0.65 | 5.14 ± 0.67 | 5.19 ± 0.69 | <0.001 |
| TC, mmol/L | 4.92 ± 0.94 | 4.96 ± 0.91 | 5.04 ± 0.95 | 5.09 ± 0.95 | <0.001 |
| TG, mmol/L | 1.61 ± 1.17 | 1.72 ± 1.23 | 1.83 ± 1.35 | 1.93 ± 1.46 | <0.001 |
| HDL-C, mmol/L | 1.42 ± 0.32 | 1.40 ± 0.31 | 1.40 ± 0.33 | 1.41 ± 0.33 | 0.001 |
| LDL-C, mmol/L | 3.55 ± 1.12 | 3.62 ± 1.15 | 3.64 ± 1.19 | 3.75 ± 1.27 | <0.001 |
| ALT, U/L | 21.00 (15.00-31.00) | 23.00 (16.00-34.70) | 23.30 (16.20-35.50) | 23.10 (16.60-35.00) | <0.001 |
| BUN, mmol/L | 5.08 ± 1.45 | 5.07 ± 1.42 | 5.08 ± 1.46 | 5.13 ± 1.49 | 0.036 |
| Scr, μmol/L | 73.47 ± 16.53 | 74.21 ± 16.18 | 74.61 ± 16.72 | 75.06 ± 21.24 | <0.001 |
| Gender, n (%) |  |  |  |  | <0.001 |
| Male | 4589 (64.57%) | 4958 (70.66%) | 5565 (71.35%) | 5298 (71.09%) |  |
| Female | 2518 (35.43%) | 2059 (29.34%) | 2235 (28.65%) | 2155 (28.91%) |  |
| Smoking Status, n (%) |  |  |  |  | <0.001 |
| Current smoker | 1302 (18.32%) | 1687 (24.04%) | 1876 (24.05%) | 2037 (27.33%) |  |
| Ever smoker | 310 (4.36%) | 315 (4.49%) | 338 (4.33%) | 287 (3.85%) |  |
| Never smoker | 5495 (77.32%) | 5015 (71.47%) | 5586 (71.62%) | 5129 (68.82%) |  |
| Drinking Status, n (%) |  |  |  |  | <0.001 |
| Current drinker | 181 (2.55%) | 265 (3.78%) | 318 (4.08%) | 387 (5.19%) |  |
| Ever drinker | 937 (13.18%) | 1059 (15.09%) | 1182 (15.15%) | 1011 (13.57%) |  |
| Never drinker | 5989 (84.27%) | 5693 (81.13%) | 6300 (80.77%) | 6055 (81.24%) |  |
| Family History of Diabetes, n (%) |  |  |  |  | 0.108 |
| No | 7007 (98.59%) | 6897 (98.29%) | 7672 (98.36%) | 7358 (98.73%) |  |
| Yes | 100 (1.41%) | 120 (1.71%) | 128 (1.64%) | 95 (1.27%) |  |
| Follow-up (years) | 2.98 (2.13-3.92) | 2.99 (2.13-3.93) | 2.99 (2.16-3.93) | 2.98 (2.15-3.92) | 0.629 |
| Diabetes in follow-up, n (%) |  |  |  |  | <0.001 |
| No | 6754 (95.03%) | 6714 (95.68%) | 7401 (94.88%) | 6958 (93.36%) |  |
| Yes | 353 (4.97%) | 303 (4.32%) | 399 (5.12%) | 495 (6.64%) |  |

Values are n (%), means or medians (quartiles)

BMI, body-mass index; FPG, fasting plasma glucose; TC, total cholesterol; TG, triglycerides; HDL-C, high-density lipoprotein cholesterol; LDL-C, low-density lipoprotein cholesterol; ALT, alanine aminotransferase; BUN, blood urea nitrogen; Scr, serum creatinine.

**Table S5. Participant characteristics across PP quartiles**

| Characteristics | Q1(n=7108) | Q2 (n=6906) | Q3 (n=7924) | Q4 (n=7367) | P-value |
| --- | --- | --- | --- | --- | --- |
| Age, years | 46.48 ± 11.86 | 49.03 ± 12.22 | 51.52 ± 14.66 | 58.80 ± 16.93 | <0.001 |
| BMI, kg/m2 | 25.18 ± 3.46 | 25.69 ± 3.32 | 25.22 ± 3.37 | 25.09 ± 3.47 | <0.001 |
| FPG, mmol/L | 5.02 ± 0.68 | 5.11 ± 0.66 | 5.18 ± 0.64 | 5.27 ± 0.66 | <0.001 |
| TC, mmol/L | 4.93 ± 0.91 | 5.00 ± 0.92 | 4.98 ± 0.94 | 5.09 ± 0.98 | <0.001 |
| TG, mmol/L | 1.79 ± 1.39 | 1.88 ± 1.40 | 1.73 ± 1.28 | 1.71 ± 1.19 | <0.001 |
| HDL-C, mmol/L | 1.38 ± 0.32 | 1.38 ± 0.32 | 1.41 ± 0.32 | 1.43 ± 0.32 | <0.001 |
| LDL-C, mmol/L | 3.59 ± 1.14 | 3.65 ± 1.20 | 3.63 ± 1.18 | 3.70 ± 1.22 | <0.001 |
| ALT, U/L | 23.50 (16.10-36.00) | 24.60 (17.00-37.70) | 22.20 (16.00-33.30) | 20.10 (15.00-29.40) | <0.001 |
| BUN, mmol/L | 4.98 ± 1.36 | 5.03 ± 1.39 | 5.11 ± 1.46 | 5.24 ± 1.58 | <0.001 |
| Scr, μmol/L | 74.56 ± 15.77 | 75.42 ± 15.83 | 74.28 ± 16.70 | 73.24 ± 22.06 | <0.001 |
| Gender, n (%) |  |  |  |  | <0.001 |
| Male | 5241 (72.99%) | 5282 (76.48%) | 5560 (70.17%) | 4327 (58.73%) |  |
| Female | 1939 (27.01%) | 1624 (23.52%) | 2364 (29.83%) | 3040 (41.27%) |  |
| Smoking Status, n (%) |  |  |  |  | <0.001 |
| Current smoker | 1882 (26.21%) | 1972 (28.55%) | 1822 (22.99%) | 1226 (16.64%) |  |
| Ever smoker | 389 (5.42%) | 285 (4.13%) | 309 (3.90%) | 267 (3.62%) |  |
| Never smoker | 4909 (68.37%) | 4649 (67.32%) | 5793 (73.11%) | 5874 (79.73%) |  |
| Drinking Status, n (%) |  |  |  |  | <0.001 |
| Current drinker | 252 (3.51%) | 330 (4.78%) | 348 (4.39%) | 221 (3.00%) |  |
| Ever drinker | 1256 (17.49%) | 1112 (16.10%) | 1123 (14.17%) | 698 (9.47%) |  |
| Never drinker | 5672 (79.00%) | 5464 (79.12%) | 6453 (81.44%) | 6448 (87.53%) |  |
| Family History of Diabetes, n (%) |  |  |  |  | 0.015 |
| No | 7051 (98.20%) | 6790 (98.32%) | 7817 (98.65%) | 7276 (98.76%) |  |
| Yes | 129 (1.80%) | 116 (1.68%) | 107 (1.35%) | 91 (1.24%) |  |
| Follow-up (years) | 3.03 (2.18-3.99) | 2.98 (2.15-3.93) | 2.96 (2.12-3.91) | 2.96 (2.13-3.87) | <0.001 |
| Diabetes in follow-up, n (%) |  |  |  |  | <0.001 |
| No | 6910 (96.24%) | 6567 (95.09%) | 7518 (94.88%) | 6832 (92.74%) |  |
| Yes | 270 (3.76%) | 339 (4.91%) | 406 (5.12%) | 535 (7.26%) |  |

Values are n (%), means or medians (quartiles)

BMI, body-mass index; FPG, fasting plasma glucose; TC, total cholesterol; TG, triglycerides; HDL-C, high-density lipoprotein cholesterol; LDL-C, low-density lipoprotein cholesterol; ALT, alanine aminotransferase; BUN, blood urea nitrogen; Scr, serum creatinine.

**Table S6. Participant characteristics across PPI quartiles**

| Characteristics | Q1(n=6925) | Q2 (n=7026) | Q3 (n=7341) | Q4 (n=8085) | P-value |
| --- | --- | --- | --- | --- | --- |
| Age, years | 46.62 ± 11.93 | 49.09 ± 12.13 | 51.88 ± 14.27 | 57.53 ± 17.43 | <0.001 |
| BMI, kg/m2 | 25.30 ± 3.51 | 25.67 ± 3.34 | 25.29 ± 3.35 | 24.94 ± 3.42 | <0.001 |
| FPG, mmol/L | 5.03 ± 0.68 | 5.10 ± 0.67 | 5.19 ± 0.64 | 5.25 ± 0.65 | <0.001 |
| TC, mmol/L | 4.94 ± 0.91 | 5.01 ± 0.92 | 5.01 ± 0.94 | 5.04 ± 0.98 | <0.001 |
| TG, mmol/L | 1.81 ± 1.40 | 1.89 ± 1.43 | 1.76 ± 1.30 | 1.67 ± 1.13 | <0.001 |
| HDL-C, mmol/L | 1.38 ± 0.33 | 1.39 ± 0.32 | 1.41 ± 0.32 | 1.43 ± 0.32 | <0.001 |
| LDL-C, mmol/L | 3.60 ± 1.15 | 3.64 ± 1.21 | 3.67 ± 1.20 | 3.65 ± 1.20 | 0.005 |
| ALT, U/L | 23.70 (16.20-36.40) | 24.70 (17.00-37.20) | 22.50 (16.00-34.00) | 20.10 (15.00-29.40) | <0.001 |
| BUN, mmol/L | 4.99 ± 1.37 | 5.03 ± 1.39 | 5.13 ± 1.48 | 5.20 ± 1.55 | <0.001 |
| Scr, μmol/L | 74.70 ± 15.86 | 75.32 ± 15.71 | 74.49 ± 17.93 | 73.10 ± 20.73 | <0.001 |
| **Gender, n (%)** |  |  |  |  | <0.001 |
| Male | 5065 (73.14%) | 5386 (76.66%) | 5150 (70.15%) | 4809 (59.48%) |  |
| Female | 1860 (26.86%) | 1640 (23.34%) | 2191 (29.85%) | 3276 (40.52%) |  |
| **Smoking Status, n (%)** |  |  |  |  | <0.001 |
| Current smoker | 1854 (26.77%) | 1993 (28.37%) | 1733 (23.61%) | 1322 (16.35%) |  |
| Ever smoker | 367 (5.30%) | 309 (4.40%) | 282 (3.84%) | 292 (3.61%) |  |
| Never smoker | 4704 (67.93%) | 4724 (67.24%) | 5326 (72.55%) | 6471 (80.04%) |  |
| **Drinking Status, n (%)** |  |  |  |  | <0.001 |
| Current drinker | 245 (3.54%) | 354 (5.04%) | 330 (4.50%) | 222 (2.75%) |  |
| Ever drinker | 1206 (17.42%) | 1161 (16.52%) | 1026 (13.98%) | 796 (9.85%) |  |
| Never drinker | 5474 (79.05%) | 5511 (78.44%) | 5985 (81.53%) | 7067 (87.41%) |  |
| **Family History of Diabetes, n (%)** |  |  |  |  | 0.033 |
| No | 6804 (98.25%) | 6906 (98.29%) | 7243 (98.67%) | 7981 (98.71%) |  |
| Yes | 121 (1.75%) | 120 (1.71%) | 98 (1.33%) | 104 (1.29%) |  |
| Follow-up (years) | 3.03 (2.18-3.99) | 2.99 (2.15-3.93) | 2.95 (2.12-3.89) | 2.96 (2.13-3.87) | <0.001 |
| **Diabetes in follow-up,** n (%) |  |  |  |  | <0.001 |
| No | 6648 (96.00%) | 6676 (95.02%) | 6976 (95.03%) | 7527 (93.10%) |  |
| Yes | 277 (4.00%) | 350 (4.98%) | 365 (4.97%) | 558 (6.90%) |  |

Values are n (%), means or medians (quartiles)

BMI, body-mass index; FPG, fasting plasma glucose; TC, total cholesterol; TG, triglycerides; HDL-C, high-density lipoprotein cholesterol; LDL-C, low-density lipoprotein cholesterol; ALT, alanine aminotransferase; BUN, blood urea nitrogen; Scr, serum creatinine.

**Table S7. Participant characteristics across SBP/DBP quartiles**

| Characteristics | Q1(n=7291) | Q2 (n=7292) | Q3 (n=7427) | Q4 (n=7367) | P-value |
| --- | --- | --- | --- | --- | --- |
| Age, years | 46.67 ± 11.92 | 49.34 ± 12.25 | 52.00 ± 14.47 | 58.03 ± 17.54 | <0.001 |
| BMI, kg/m2 | 25.31 ± 3.50 | 25.66 ± 3.33 | 25.25 ± 3.34 | 24.94 ± 3.44 | <0.001 |
| FPG, mmol/L | 5.03 ± 0.68 | 5.11 ± 0.66 | 5.20 ± 0.64 | 5.25 ± 0.65 | <0.001 |
| TC, mmol/L | 4.95 ± 0.91 | 5.01 ± 0.92 | 5.01 ± 0.95 | 5.05 ± 0.98 | <0.001 |
| TG, mmol/L | 1.81 ± 1.39 | 1.88 ± 1.44 | 1.75 ± 1.28 | 1.66 ± 1.13 | <0.001 |
| HDL-C, mmol/L | 1.38 ± 0.33 | 1.39 ± 0.32 | 1.41 ± 0.32 | 1.43 ± 0.32 | <0.001 |
| LDL-C, mmol/L | 3.61 ± 1.15 | 3.64 ± 1.21 | 3.66 ± 1.19 | 3.66 ± 1.20 | 0.031 |
| ALT, U/L | 23.80 (16.30-36.60) | 24.40 (17.00-37.00) | 22.30 (16.00-33.25) | 20.00 (15.00-29.00) | <0.001 |
| BUN, mmol/L | 4.99 ± 1.37 | 5.04 ± 1.40 | 5.13 ± 1.48 | 5.21 ± 1.56 | <0.001 |
| Scr, μmol/L | 74.75 ± 15.82 | 75.31 ± 15.79 | 74.35 ± 17.82 | 73.01 ± 21.18 | <0.001 |
| **Gender, n (%)** |  |  |  |  | <0.001 |
| Male | 5354 (73.43%) | 5554 (76.17%) | 5189 (69.87%) | 4313 (58.54%) |  |
| Female | 1937 (26.57%) | 1738 (23.83%) | 2238 (30.13%) | 3054 (41.46%) |  |
| **Smoking Status, n (%)** |  |  |  |  | <0.001 |
| Current smoker | 1970 (27.02%) | 2040 (27.98%) | 1731 (23.31%) | 1161 (15.76%) |  |
| Ever smoker | 384 (5.27%) | 312 (4.28%) | 281 (3.78%) | 273 (3.71%) |  |
| Never smoker | 4937 (67.71%) | 4940 (67.75%) | 5415 (72.91%) | 5933 (80.53%) |  |
| **Drinking Status, n (%)** |  |  |  |  | <0.001 |
| Current drinker | 272 (3.73%) | 350 (4.80%) | 335 (4.51%) | 194 (2.63%) |  |
| Ever drinker | 1266 (17.36%) | 1189 (16.31%) | 1034 (13.92%) | 700 (9.50%) |  |
| Never drinker | 5753 (78.91%) | 5753 (78.89%) | 6058 (81.57%) | 6473 (87.86%) |  |
| **Family History of Diabetes, n (%)** |  |  |  |  | 0.010 |
| No | 7163 (98.24%) | 7166 (98.27%) | 7338 (98.80%) | 7267 (98.64%) |  |
| Yes | 128 (1.76%) | 126 (1.73%) | 89 (1.20%) | 100 (1.36%) |  |
| Follow-up (years) | 3.03 (2.18-3.99) | 2.98 (2.15-3.93) | 2.96 (2.12-3.89) | 2.96 (2.13-3.87) | <0.001 |
| **Diabetes in follow-up,** n (%) |  |  |  |  | <0.001 |
| No | 6998 (95.98%) | 6921 (94.91%) | 7056 (95.00%) | 6852 (93.01%) |  |
| Yes | 293 (4.02%) | 371 (5.09%) | 371 (5.00%) | 515 (6.99%) |  |

Values are n (%), means or medians (quartiles)

BMI, body-mass index; FPG, fasting plasma glucose; TC, total cholesterol; TG, triglycerides; HDL-C, high-density lipoprotein cholesterol; LDL-C, low-density lipoprotein cholesterol; ALT, alanine aminotransferase; BUN, blood urea nitrogen; Scr, serum creatinine.

Factors influencing risk of mortality analyzed by univariate Cox proportional hazards regression analysis

**Table S8.** **Factors influencing incident diabetes analyzed by univariate Cox proportional hazards regression.**

| Variable | Characteristics | HR (95% CI) | P-value |
| --- | --- | --- | --- |
| SBP (10 mmHg) | 14.54 ± 1.35 | 1.27 (1.23, 1.31) | <0.0001 |
| DBP (10 mmHg) | 8.99 ± 1.02 | 0.93 (0.89, 0.98) | 0.0049 |
| MAP (10 mmHg) | 10.84 ± 0.83 | 1.17 (1.11, 1.24) | <0.0001 |
| PP (10 mmHg) | 5.56 ± 1.66 | 1.22 (1.19, 1.26) | <0.0001 |
| PPI | 0.38 ± 0.09 | 25.47 (14.65, 44.26) | <0.0001 |
| SBP/DBP | 1.64 ± 0.25 | 3.06 (2.55, 3.68) | <0.0001 |
| Age, years | 51.53 ± 14.84 | 1.03 (1.03, 1.04) | <0.0001 |
| BMI, kg/m2 | 25.29 ± 3.41 | 1.12 (1.11, 1.14) | <0.0001 |
| FPG, mmol/L | 5.15 ± 0.66 | 6.45 (5.99, 6.95) | <0.0001 |
| TC, mmol/L | 5.00 ± 0.94 | 1.15 (1.09, 1.21) | <0.0001 |
| TG, mmol/L | 1.78 ± 1.32 | 1.17 (1.15, 1.20) | <0.0001 |
| HDL-C, mmol/L | 1.40 ± 0.32 | 0.98 (0.84, 1.15) | 0.8081 |
| LDL-C, mmol/L | 3.64 ± 1.19 | 0.97 (0.93, 1.01) | 0.1800 |
| ALT, U/L | 22.60 (16.00-34.00) | 1.00 (1.00, 1.01) | <0.0001 |
| BUN, mmol/L | 5.09 ± 1.46 | 1.08 (1.05, 1.12) | <0.0001 |
| Scr, μmol/L | 74.35 ± 17.82 | 1.00 (1.00, 1.00) | 0.6041 |
| Gender, n (%) |  |  |  |
| Male | 20410 (69.48%) | 1.0 |  |
| Female | 8967 (30.52%) | 0.99 (0.88, 1.10) | 0.7923 |
| Smoking Status, n (%) |  |  |  |
| Current smoker | 6902 (23.49%) | 1.0 |  |
| Ever smoker | 1250 (4.26%) | 1.25 (1.02, 1.54) | 0.0323 |
| Never smoker | 21225 (72.25%) | 0.72 (0.64, 0.80) | <0.0001 |
| Drinking Status, n (%) |  |  |  |
| Current drinker | 1151 (3.92%) | 1.0 |  |
| Ever drinker | 4189 (14.26%) | 0.81 (0.61, 1.06) | 0.1238 |
| Never drinker | 24037 (81.82%) | 0.75 (0.58, 0.96) | 0.0223 |
| Family History of Diabetes, n (%) |  |  |  |
| No | 28934 (98.49%) | 1.0 |  |
| Yes | 443 (1.51%) | 1.42 (1.04, 1.95) | 0.0274 |

**Table S9. Discriminative performance of BP Parameters and the incident diabetes**

| **Variable** | **AUC** | **95%CI low** | **95%CI upp** | **Specificity** | **Sensitivity** |
| --- | --- | --- | --- | --- | --- |
| SBP (10 mmHg) | 0.5875 | 0.5730 | 0.6020 | 0.5958 | 0.5284 |
| DBP (10 mmHg) | 0.5077 | 0.4918 | 0.5236 | 0.7840 | 0.2639 |
| MAP (10 mmHg) | 0.5402 | 0.5245 | 0.5558 | 0.8029 | 0.2781 |
| PP (10 mmHg) | 0.5741 | 0.5593 | 0.5890 | 0.7955 | 0.3090 |
| PPI | 0.5626 | 0.5477 | 0.5775 | 0.7295 | 0.3600 |
| SBP/DBP | 0.5625 | 0.5475 | 0.5774 | 0.7938 | 0.2948 |

**Table S10. Associations between BP Parameters and the incident diabetes Using Non-imputed Data**

| Exposure | Model I (HR,95%CI) P | Model II (HR,95%CI) P | Model III (HR,95%CI) P |
| --- | --- | --- | --- |
| **SBP (10 mmHg)** | 1.27 (1.23, 1.31) <0.0001 | 1.13 (1.09, 1.17) <0.0001 | 1.09 (1.05, 1.13) <0.0001 |
| **DBP (10 mmHg)** | 0.93 (0.89, 0.98) 0.0049 | 0.96 (0.91, 1.01) 0.0802 | 1.01 (0.96, 1.06) 0.7368 |
| **MAP (10 mmHg)** | 1.17 (1.11, 1.24) <0.0001 | 1.06 (1.00, 1.12) 0.0472 | 1.07 (1.02, 1.13) 0.0094 |
| **PP (10 mmHg)** | 1.22 (1.19, 1.26) <0.0001 | 1.12 (1.08, 1.15) <0.0001 | 1.06 (1.03, 1.10) 0.0002 |
| **PPI** | 25.47 (14.65, 44.26) <0.0001 | 6.37 (3.51, 11.57) <0.0001 | 2.47 (1.36, 4.46) 0.0028 |
| **SBP/DBP** | 3.06 (2.55, 3.68) <0.0001 | 1.93 (1.58, 2.36) <0.0001 | 1.38 (1.13, 1.70) 0.0019 |

Model I: we did not adjust other covariates.

Model II: we adjust age, gender and BMI.

Model III: we adjust variables in Adjust II+ FPG, TC, TG, ALT, BUN, smoking status, Drinking status, Family history of diabetes. HR Hazard Ratio, CI Confidence Interval.

**Table S11. Associations of BP Parameters with Incident Diabetes Based on FPG Criteria (≥7.0 mmol/L, Excluding Self-reported Cases)**

| Exposure | Model I (HR,95%CI) P | Model II (HR,95%CI) P | Model III (HR,95%CI) P |
| --- | --- | --- | --- |
| **SBP (10 mmHg)** | 1.27 (1.23, 1.32) <0.0001 | 1.15 (1.11, 1.20) <0.0001 | 1.22 (1.13, 1.33) <0.0001 |
| **DBP (10 mmHg)** | 0.98 (0.93, 1.04) 0.5092 | 0.98 (0.93, 1.04) 0.5624 | 1.07 (0.95, 1.20) 0.2527 |
| **MAP (10 mmHg)** | 1.22 (1.15, 1.30) <0.0001 | 1.10 (1.04, 1.17) 0.0014 | 1.24 (1.09, 1.40) 0.0011 |
| **PP (10 mmHg)** | 1.20 (1.16, 1.24) <0.0001 | 1.12 (1.08, 1.16) <0.0001 | 1.14 (1.05, 1.23) 0.0013 |
| **PPI** | 16.95 (9.25, 31.04) <0.0001 | 6.10 (3.18, 11.71) <0.0001 | 5.19 (1.23, 21.87) 0.0247 |
| **SBP/DBP** | 1.02 (1.01, 1.03) <0.0001 | 1.01 (1.00, 1.02) 0.0014 | 1.02 (1.01, 1.03) 0.0010 |

Model I: we did not adjust other covariates.

Model II: we adjust age, gender and BMI.

Model III: we adjust variables in Adjust II+ FPG, TC, TG, ALT, BUN, smoking status, Drinking status, Family history of diabetes. HR Hazard Ratio, CI Confidence Interval.

**Figure S1.** Distribution of BP Parameters. A: SBP; B: DBP; C: MAP; D: PP; E: PPI; F: SBP/DBP.

**Figure S2.** The incidence rate of diabetes of age stratification by 10 intervals. It shows that the incidence rate of diabetes in participants was higher in man than in women, regardless of age group, but in age ≥70 years old， the rate in women was higher than it in men. It was also found that the incidence rate of diabetes decreases with increasing age for both men and women.
